# Medical Reform

**Published:** 1845-01

**Authors:** 


### MEDICAL REFORM.

I. Article on Medical Reform in Quarterly Review. Volume Sixty-seven. 1841.

II. The Touchstone of Medical Reform. By *Joseph Henry Green.* 1841.

III. A Statement by the Society of Apothecaries. 1844.

IV. A Bill for the better Regulation of Medical Practice throughout the United Kingdom. 1844.

V. An Address of the Society of Apothecaries. 1844.

VI. A Manifesto by the Medical and Surgical Association of the Borough of Marylebone. 1844.

VII. A Speech delivered at Derby. By *Charles Hastings*, M.D., President of the Council of the Provincial Medical and Surgical Association. 1844.

VIII. A Letter from Charles Carter, Esq. to the North of England Medical Association. 1844.

IX. An Exposition of the Laws which relate to the Medical Profession, with an ample Analysis of Sir James Graham's Bill. By *John Davies*, M.D. 1844.

X. Sir James Graham's Bill repudiated. By *John Thomson*, M.D. 1844.

A movement in the medical world equal to the present has never been witnessed. It is no partial result of an objectless agitation, but the throe of a large and important body, wounded in its most vital parts. The *vis inertia* of the Profession has been overcome, and men are called out from that tranquillity and retirement which they love, and which in many instances they have well earned; for, it is to be observed, that the present opposition is joined in often times as keenly, although with more discrimination, by several senior practitioners whose prospects it can little affect, but who feel actuated by a laudable *esprit de corps*, as it is by those who have not long entered the Profession, and who believe their future prospects to be seriously menaced by the Bill. It is curious to observe the workings of the human mind throughout the thirty thousand medicos in this mighty empire, while agitating this question! A good many of the high aristocratic order, believing that their own pecuniary interest is but little concerned in the event, look on in apathy, and think little on the subject. Another good sprinkling of the Pures—medical and chirurgical, calculating on indirect gain, join in the cry against the project of the Home Secretary in hopes of increased popularity. But the great body of General Practitioners alarmed, and not without reason, that their income will be diminished, are up in arms against the "free-trade" in Physic which our legislators are preparing for us. Thus, self-interest is the grand propelling power, after all, verifying the sentiment of the poet—

"Self-love and social are the same."

The Minister acted prudently and fairly in allowing so long an interval to elapse between the proposition of the measure and any attempt being made to carry it; seeing that, with his power in the house, and the apparent concord of all parties there in its approval, he might have carried it at a short notice, before it could be effectually opposed, or even properly understood by the profession. The formidable opposition which has been generated during this delay is not, however, one whit stronger than the crisis demands; for the various provisions of the Bill have been framed with far too much connectedness of purpose and deliberation to be withdrawn, or even modified, unless a really imposing power be arrayed against them. Any one who will be at the pains of perusing the two first documents named at the head of this article, (and they are well worthy of a very attentive perusal,) will see shadowed forth in no indistinct manner, more than three years since, not only the principles but the practical provisions of this measure. Suggested then by such influential members of the Profession as are the authors of these tracts, and adopted by the Ministry after so deliberate a consideration, and with so few additions, we may be certain they will be relinquished with difficulty, and at most but partially.

We have much greater faith in the efficacy of the opposition than we had awhile back, inasmuch as it has become more moderated in its tone and more reasonable in its object. At first, the Profession, which had been so long demanding the representative system as the bread of its corporate existence, stood aghast when a stone in the shape of the present bill was offered it, and panic-struck at the deprivation of status and irruption of the unlicensed which seemed imminent, became frighted from its propriety, and determined upon being satisfied with nothing short of the unconditional rejection of so obnoxious a measure. Even now, when sufficient time for mature reflection has elapsed, the power which unanimity would confer is withheld by a section of ultra-opponents, whose numbers, however, we feel convinced must diminish in proportion as inquiry into the capabilities of the measure and wants of the profession progresses. Suppose even the Profession possessed power enough to prevent the introduction of the present bill at all, would it act prudently in putting it into action? We think most decidedly it would not. That a reform in our medical institutions has become essential to their well-being, is either a fact, or is believed to be one, by so preponderating a majority of their members, that tranquillity and harmony can never be expected until it has been achieved. We have seen the utter failure on the part of all those who have hitherto attempted it, in producing a measure either satisfactory to the profession or palatable to the legislature: and if, after such constant urging as he has been plied with, a minister of the administrative talents of Sir James Graham prepares a measure with great care and deliberation, and yet is unable to carry it in even a modified form, in a house where his influence is so great, we shall be curious to see the member who is sanguine or foolish enough to attempt anything on behalf of so impracticable a matter for the next twenty years to come, at least. In the mean time we must go on in the same unsettled and unsatisfactory state, patching up this corporation, and fashioning that one, according to the necessities of the moment and the urgency of the demand.

The opposition has not only been injudiciously exclusive, but also somewhat unfairly conducted. Charges have been brought against the framers of the bill of a deliberate design to inflict a deadly blow upon the well-being of the general practitioner, which nothing but an exaggerated and one-sided view of its clauses will justify: and even so pitiful a motive as a desire of revenge, in consequence of the measure of success which attended the medical opposition to the New Poor Law, has been assigned as actuating the minister in the introduction of a measure which is at least of as much importance to the public at large as to the profession. We are not disposed to join our brethren in congratulating themselves upon the possession of the powerful co-operation of the *Times* in the agitation of this question. Our cause ought to be strong enough and just enough to stand upon its own basis, without enlisting party-writing upon its behalf; and it is surely derogatory to the Profession to be made the cat's-paw of any journal, however eminent. The *Times* participated in the general ignorance of the public press as regards medical matters, until the fact of Sir James Graham having come into collision with the profession upon this question, induced it to employ an able pen in the hope of adding still farther to the odium it has so long and so perseveringly sought to attach to all this statesman's acts.

It is not our intention to examine the Bill clause by clause, for its analysis has been so completely and repeatedly undertaken, by both its opponents and advocates, that the Profession ought to be tolerably well acquainted with its various provisions by this time. We will confine our attention to noticing its principal defects and omissions, believing, as we stated in our last, that a few strokes of the pen might render this much-abused measure a great benefit to the community, as well as to the profession. Its two plague-spots are the faulty construction of the Council, and the omission of a penalty upon non-registered practitioners; and to the consideration of these points we will first address ourselves.

The *Constitution* of the *Council of Health and Education* is beyond all comparison the most important feature of the measure, and yet the hundreds of petitions and resolutions, which have been drawn up in various parts of the country, contain scarcely any suggestions as to its composition, but are almost exclusively occupied in complaints against quackery. The powers and duties of the Council, as respects the various objects of State Medicine, we are not informed of, but in all matters regarding the profession it will be the supreme and absolutely controlling body, possessed of the most ample powers for the direction of the course of medical education, the sums to be paid for it, the examinations of candidates, the registration of practitioners, and even determining which grades of the profession shall fill certain offices. We do not say that the possession of these powers is not essential to the establishment of a system of uniformity, and that connexion with Government which is now generally admitted to be the most feasible project for the removal of anomalies, healing differences, and contributing to improvement; but is it not of the last importance that a body invested with such despotic powers should be so constituted as to afford the best chance of their being wielded to the advantage of the public and of the profession. This can only be accomplished by a *representative system*, the public on the one hand and each branch or grade of the profession upon the other, having its voice in the Council. The Bill in fact acknowledges a principle of representation, but carries it out in a most partial and unfair manner. Each University, and each College of Physicians and College of Surgeons in the United Kingdom, furnishes a member of the Council, while the Crown is represented by the Home Secretary, President *ex officio*, and six of his nominees ; and in this way are the eighteen places, of which it is proposed it should consist, filled up. But, when it is asked what part the general practitioners, who constitute nineteen-twentieths of the profession, and to regulate whose interests this Council is chiefly formed, are to take in the deliberations of this body, the reply is, none whatever, they are unrepresented there! It may be said that some of the six nominees of the Crown may be selected from this body, and they probably would be so; but it will never do for the great mass of practitioners to depend upon any mere chance of this kind, for, as far as the Bill enacts, these persons need not be members of the profession at all. The general practitioners must not seek to have a voice in the Council as a favour, at the will of the Minister for the time being, but must demand it firmly as an indisputable right. Seven members of the Council are more than the Crown should furnish, for there is some risk, owing to the frequently unavoidable absence of the members connected with the Universities and Colleges, of the Institution degenerating into a mere board of government officials, exerting an undue influence over the profession. Three of these at least should be resigned to the representatives of the General Practitioner, one for each division of the Kingdom.

The construction of this Council, we repeat, is of much more importance than is generally supposed, and the subject should be embraced in every petition to Parliament. If more allusion to this topic be not made in the resolutions at public meetings, it will be supposed by the Home Secretary that little objection to the proposed construction exists, and it may be crammed down the throats of the Profession, before they are aware of the poison they are swallowing.

*Penal Clauses.*—A very natural feeling of alarm and indignation has been manifested by the profession upon the announcement of the intended abandonment of all attempts at direct penal restriction of unlicensed practitioners ; and although, perhaps, the evil effect this would have upon the medical body has been exaggerated, its tendency to injure the public can scarcely be doubted by those who will take the pains to think upon the matter. The absence of any provision of this kind, in the present Bill, although a rather curious reply to the numerous petitions for additional protection which of late years have been presented, is not very surprising, considering that opinions in favour of this free-trade system of physic are held by more than one eminent member of the profession itself. The

Quarterly Reviewer, for example, declares he cannot see the propriety, after having pointed out to the public who are qualified practitioners, of still farther interfering and preventing any one who choses in preference to consult those that are not so. Moreover, he adds, there are cases, do what you will, you cannot reach.

> "When the resources of skill and science fail, the instinct of self-preservation will lead many sufferers to look for other aid; and the honest and well-educated practitioner will always have to contend, not only with the St. John Long's of the day, but with those among his own brethren who do not partake of his anxiety to avoid making promises which cannot be fulfilled. There are, in fact, no more offensive impostors than those who march under the banner of the true faith, and we suppose that even the most sanguine petitioner against quackery will not expect that such as these can be extinguished by an act of Parliament."

This is to argue upon the exceptions of the case, and to leave out of view the real evils to be provided against. Few indeed are foolish enough to believe that Quackery can or ever will be suppressed, or are desirous of entering upon a crusade for its extirmination ; but this affords about as good a reason for the abandonment of all attempts at confining its ravages within some limits, as the persistence of the crime of thieving, notwithstanding the infliction of imprisonment upon its detection, does for the abolition of that punishment. To the unfortunate being who has exhausted all the appliances of legitimate medical skill, it would indeed be cruel to deny the desperate hope and melancholy satisfaction of seeking aid elsewhere; and, as for the black-sheep of our body, we must be content with affixing the seal of professional disapprobation upon their proceedings. Nay, more, yielding thus much to those who are so desirous of the liberty of the subject in this matter, we may state we feel no anxious desire for a law which would restrain Lord this, Lady that, or any of the well-lined-purse and heated-imagination-tribe from seeking out and consulting the whole herd of empirics simultaneously or in succession. But is it not childish to point to the consolation which a few desperate cases derive from the attentions of those who will promise them all and everything, as long as there is a penny to pay for it, while you neglect the vast numbers whose maladies have become aggravated into danger, or prolonged in their continuance, through their having inadvertently fallen into the hands of the uneducated? So, too, because a few foolish persons in the upper classes, having more money than wit, and more indolence of body and mind than either, cannot (and need not, as their position in society furnishes them with the means of detection of impostors if they choose to employ them), be prevented consulting and encouraging charlatanism, is the great mass of the humbler classes of the community, possessed of no means of distinguishing the qualified from the unqualified, and usually crediting a man's pretensions just in proportion to his boldness in asserting them, to be allowed to be preyed upon by pretenders, however ignorant, and the protection denied them which this very Bill specially provides for the Union pauper and the felon? Yes, the honest artizan or independent labourer has not the same chance afforded him in the qualifications of his attendant, during an illness whose duration may decide the prospects of himself and family for life, as has the wretched pauper or convicted thief! To carry crotchets like these into practical operation is no laughing matter.

Mr. Green, in some passages we extract from his pamphlet, discourses a sounder philosophy than his brother councillor.

"It cannot be doubted that it is the duty of a Government to provide for the well-being of social man, in his relations and duties of citizen, by supplying the requisites for his moral cultivation, his social security, and his health; and it is rather the consideration of the practical difficulties, than any doubt of the principle of interference, that would prevent my urging the Legislature to withdraw its countenance, accorded under a specious and mis-used plea of liberty, for the knavish quack, and to protect, by penal laws, his patients, who vindicate their civil rights at the expense of being robbed, maimed, and poisoned. Certainly it is disgraceful to the country that its Government should derive a revenue from so unholy a source as that of patents granted for secret remedies and pernicious nostrums."

And again:—

"But in considering farther the functions which might be advantageously entrusted to a supreme State Council for Medical Affairs, it may well deserve consideration, whether the duty of taking *cognizance of the practice of unqualified persons* might not properly be committed to it; and whether, with this view, and as far as this specific object is concerned, it ought not to form a tribunal invested with powers judicial and penal. It is self-evident that the interests of the profession and the public require protection of some kind from ignorant and dishonest pretenders, and though it may be impossible to prevent the multitude having recourse to quacks, nostrum-mongers, and the impudent tribe of vaunters and venders of secret remedies, yet it can scarcely be deemed other than a duty of the Government to restrain, as far as possible, the practices of those who usurp the name and functions of regular practitioners. And though a prohibitory law might be evaded in some, or even in all instances, I cannot but think that there would be a propriety in giving the stamp of illegality to irregular practices, even if only with a view to the possible advantages of its moral influence in checking them; and I should not be without hope that a more effectual restraint might be imposed; if, on the one hand, each corporate body had its members duly registered,—if such designations were accredited, and appropriated to regular practitioners, as *Doctors of Medicine, Masters of Surgery, and Licentiates of Medicine aud Surgery, and of Midwifery,*—if all prescriptions were required to have affixed to them the name and designation of the writer—if the operations of surgery and midwifery could only be performed by legally-qualified practitioners,—if the preparation and sale of medicines were confined to licensed persons:—and then, on the other hand, if the tribunal, proposed above, were armed with powers of summary conviction and punishment, on proof that the offender had assumed the designation or exercised the functions of a legally-qualified practitioner, and *had practised unlawfully for gain.*"

In reference to the *practicability and utility* of enforcing any penal measures, it must be borne in mind that there are two different classes of unlicensed practitioners, proceedings against whom would probably be attended with different measures of success. There is the genuine *quack doctor,* boasting empiric or undoubted charlatan, known as such by the public, and carefully avoided by the great majority, who has infested all countries at all times, and grappling with whom, we grant, would be a matter of difficulty, and must seldom be attempted. But the persons from whom the public and profession alike chiefly suffer are those who assume all the functions of a regular practitioner of medicine, without being in the possession of any of his qualifications—a far more numerous and dangerous class than the quacks properly so called, since they are more difficult of detection, and would still be employed by many, if detected, on account of the greater cheapness of their charges, which naturally results from the absence of expenditure for their qualification and their equivocal position in society. It is against this class penal laws should especially be directed, and to those who say their repression is impossible, we reply, at least let the experiment be fairly made, which it never yet has been. The fact of the College of Physicians and Apothecaries having been invested with powers for the suppression of irregular practice, and not having succeeded in so doing, says but little. The prosecutions undertaken by the College of Physicians did doubtless restrain many unqualified persons, but the greater number were instituted at a period when the supply of properly-educated men was by no means adequate to the public requirements, while they were too often directed against persons as well educated as the prosecutors themselves.

As to the Apothecaries' Act, its provisions are so completely inadequate to the end proposed, that it seems to have been framed rather for the purpose of bringing restrictions into disrepute than for facilitating their application. The Society would have done a good service to the public had they furnished, in their "Statement," a tabular view of the particulars of the whole of the prosecutions they have instituted; setting forth the precise number of actions, the dates of their commencement and termination, the expenses incurred, and the *description of persons affected by them.* The difficulty of proof of attendance is so great, the delay in bringing the offender to justice so considerable, the chances of a verdict being obtained so uncertain, and the expenses incurred are so enormous (averaging above £300 in six cases), that a direct premium to offenders may be said to be almost held out. Worst of all, this unwieldly machinery has been set on foot, we believe, in the majority of instances, not against the ignorant pretender, but against educated gentlemen, members of some other of the medical corporations, but not possessing a license from the Society of Apothecaries, and who had probably proved themselves troublesome neighbours to unsuccessful rivals.

The Council of the College of Surgeons has frequently been pointed to as neither possessing or desiring to possess a restrictive power. A singular boast indeed, whether we consider the dignity of the branch of the profession over which it presides, or the safety of the public! Do we not see the most ignorant pretenders assume that which should be a honourable appellation, indicative to the public of certain attainments in its possessor, and even acquire by such assumption a right to exact money for attendance and medicines which the Apothecaries' Act would otherwise (for with all its faults it has had some effect both in the direct and indirect restriction of pretenders) prevent the enforcement of? Is it not notorious, too, that any operation, however grave, may be undertaken, and any malady, which by latitude of interpretation can be termed surgical, may be treated by persons who have received no kind of professional education whatever, and who, if they ever acquire any tact in the treatment of disease, do so at the expense of those who are luckless enough to first fall into their grasp?

The amount of injury which will be inflicted upon the profession by permitting the uneducated or half-educated (for numbers who by the present regulations are compelled to qualify themselves will be content then with a mere smattering picked up as assistants or shop-boys,) to engage in the practice of medicine, is not to be measured alone by the diminution of emoluments which will result, but chiefly by the loss of *caste*, so to speak, it must entail. The regular practitioners, unprotected by the institutions to which they belong, will become more or less confounded in the general estimation with the pretenders, and suffer in consequence of their mal-practices ; for the popular idea of the medical and and other professions has always been derived rather from the errors and delinquencies of interlopers and disreputable members, than from the actions of the well-disposed and honourable portions of them. Then, again, the removal of restrictions which have been once imposed, is a tacit admission of their injustice or inappropriateness, and must contribute to the elevation of persons liberated from their operation in the public estimation.

But, it will be replied, the Bill offers an indirect opposition to the unlicensed practitioner, by refusing to allow him to hold any public appointment, to enrol his name upon the registry, or to sue in a court of law. Little must he know of the real state of the case who believes that persons of this class are usually ambitious of holding public appointments, which would soon reveal their ignorance, that patients will give themselves the trouble, or can often have the opportunity, of searching a voluminous registry, or that the withholding the very questionable privilege of going to law with their patients, will operate in any other way than in preventing them giving them credit. Sir James Graham professes to desire rather to encourage and reward the legitimate practitioner, than to suppress and punish the pretender ; but, before he will be able to carry this Good-Boy Principle out, he must have something a little more tempting to offer than Union-doctorships and the like.

The proof of qualification, as stated in the Bill, should be *registration ;* but this, to be of any service, must be *compulsory,* without which it is no boon whatever. Any person presuming to practise medicine or surgery without having his place on such registry, should be made liable to fine or imprisonment, upon summary conviction before a magistrate, it being a special duty of the secretary or registrar of the Council of Health to prefer informations of such illegal practices. Public and professional opinion would alike prevent this clause being oppressively worked in the exceptional cases before mentioned. The case of the *chemists and druggists* presents the only obstacle that we know of to the effectual adoption of the above proceeding. Not but that, if this class of persons visited and attended patients, perfectly unqualified as they are for any such duty, there should be the slightest hesitation in putting the penalties in force against them, as indeed none would desire more anxiously than the truly respectable members of their own body. But we believe that proceedings of this kind could not be put into force against what is termed *counter-practice,* the giving incidental medical advice to the purchasers of drugs. And, indeed, we can scarcely see the justice of requiring this on the part of the profession, however useful it might prove, if practicable, to the public, unless registered practitioners also agreed to abandon the practice of selling drugs —a clear invasion of the territory of the druggist, and one which has contributed much to confound the two classes of persons in the eye of the public. We believe there is no hope of such compromise being entered into, and in some respects it is advantageous to the public that it should not be. If the chemist, on the one hand, does some harm by venturing to advise on a subject he does not understand, the open shops of surgeons, on the other, confer a greater benefit upon the humble classes in large towns than many are aware of, numbers of whom, if these were closed, would be driven to have recourse to the unqualified, or to charitable institutions. Young men of good abilities and education, but without a large circle of acquaintances, are often the proprietors of these, and are willing to bestow considerable attention to the cases which fall in their way for a very moderate remuneration, in the hopes that the reputation they acquire may lead, as it so frequently does, to better things. A shop seems a kind of public property into which many a patient will at once walk, who cannot muster up courage to disturb the quietude of the closed private door, be the brass-plate ever so invitingly large.

Let us view the proposed Bill, in the last place, in its relations to the *existing Medical Corporations*. *Of the College of Physicians* we have nothing to observe. The prospect of repairing their dilapidated finances by a share in the examination fees, will probably act as a sufficient salvo for any loss of dignity the projected superintendence of their arrangements by the Council may seem to inflict.

The governing powers of the *College of Surgeons* have come into an unfortunate, and what we must consider an alarming, collision with the great body of its members, just at the very period when unanimity and confidence are most required. The precipitate grant of and mode of working out the new Charter, have been followed by the deepest dissatisfaction, which even its abrogation can scarcely allay. The intention of both this document and of the new Bill is evidently the creation of two grades in the profession, and it is not to this intention, but rather to the injudicious manner it has been sought to be carried out, that rational objection can be offered. The requirements of society, and the convenience of candidates, necessitate that admission into the ranks of the profession be attainable by the *minimum* of qualifications which will ensure respectability of attainments, and capability of future improvement, should opportunities offer. But surely we must not stop here. Those whose pecuniary means or greater talents allow them to pursue a more extended course of study, or to dive deeper into the penetralia of our science, must be encouraged to higher aims, which they can only be, by being assured that their acquirements will be submitted to a more severe test, and that the probability of their eventually obtaining profitable appointments will bear some relation to the pains they have taken to qualify themselves for the adequate fulfilment of their duties. Acknowledging the principle of different grades put forward by the Council of the College, we cannot approve of the mode in which they have sought to bring it into practical operation. In the first place, the change in the constitution of the body has been effected far too suddenly. It should have been purely prospective. A certain form of examination should have been announced, to which every person, whether member or not, aspiring to the dignity of the fellowship must submit, with the exception, perhaps, of the senior members, who might be admitted as an act of courtesy due to their standing. The Council has chosen to arbitrarily select four hundred members as the senior fellows, without submitting them to any test whatever, or demanding the payment of any fee, the former of which all future aspirants must undergo, and the latter all successful candidates must produce. The principle which has been followed in the selection is not to be discovered; for, although eminent names are necessarily included, others of not the slightest note are there to be found; although some of the seniors of the profession are on the list, so is the member of yesterday; although the great bulk, especially of the first batch, are pure surgeons and lecturers, some of these are left out, and there is a sufficient sprinkling of general practitioners, suffering too the *taint* of midwifery, to show that exclusiveness of this kind was found unwise or impracticable. An arbitrary proceeding like this, where one member is raised above the head of his neighbours, without why or wherefore, confers no honour upon those selected, while it inflicts an unmerited degradation upon those who have been omitted.

We cannot agree with Dr. Davies, that the new Bill will remedy the defective operation of this Charter. The 18th and 28th clauses do not seem to confer upon any member, as he supposes they do, the right of registering upon the same footing as the newly-elected fellow. We believe it will be found consistent with the tenour of the Act, in harmony with which the Charter has been prepared, that the Fellow shall register as "Surgeon," and the member, holding also the Apothecaries' license, as only "Licentiate in Medicine and Surgery." If we are correct, we much mistake if the great body of the members of the College do not peremptorily insist upon admission forthwith as fellows, thus annulling at once the practical effect of the Charter, which, however beneficially it might have operated, both for the public and the profession, if its provisions had been more liberally carried out, can now be only looked upon as an instrument for the degradation of the great body of the members, in the arbitrary elevation, by a distinctive title readily understood by the public, of less than a twentieth portion of their number, without any ascertained right or qualification whatever.

Supposing, however, that the selection of Fellows had been made in a just and wise manner, we protest entirely against the *confining the election of the governing body of the College to their number*. Every Member has as great an interest in the good government of the Institution as the Fellows can have. Science, especially Medical Science, is a bad theatre for Monarchy, Oligarchy, or Aristocracy! It will never flourish under such governments. If ever profession required or deserved that its members at large should have a voice in the election of its ruling council, it is that of Medicine. The people have a voice in the return of members to Parliament, and surely the commonalty of medical science should have a voice in the election of their own representatives. What, it will be remarked, give the elective franchise to members as fellows? We say, why not? A ten-pound freeholder has as good a vote for the return of a member of Parliament as a five-hundred-pound freeholder; and, is it not insulting to the medical profession that its members should not possess an equal vote with its fellows or other functionaries? If the Members of the College elected their own Council at periodical intervals, the task of selecting their Representative in the Council of Health might possibly be advantageously left to this body ; or it might, upon so important an occasion, refer two or more names to the final decision of the members at large.

Mr. Green objects to the members participating in the election of the Council upon grounds we should hardly have expected from the possessor of so philosophical a mind as his. After suggesting the establishment of an elective body still more restricted than the recent fellowship, inasmuch as they would be precluded from the practice of pharmacy and midwifery, he continues—

"We cannot but anticipate the probability of its not satisfying those who are desirous of obtaining a share in the management of our college, and whose especial aim it is to give to the mode of election into the Council a more popular character. It would be idle to attempt to convince those who have been imposed upon by the unmeaning term *self-election,* which is only a false designation for the mode prescribed by the Charter for supplying vacancies in the Council by *selection,* that popular and open elections, besides being fraught with all the evils of faction and intrigue, would not be compatible with the permanent interests of a College, an institution for the promotion of science, or of the profession."

We do sincerely hope that the ruling powers of the College will shortly be prepared to meet the great body of the members in a more conciliating spirit, and a more just appreciation of their mutual interests, than this passage displays. It behoves an Institution, so dependent upon the good opinion of the profession, to be most careful in its movements. The proposition contained in the "Manifesto" of the general practitioners of Marylebone, for the formation of "A College of General Practitioners in Medicine, Surgery, and Midwifery," which awhile since would have excited no more attention than have numerous similar projects, will be responded to, we doubt not, most extensively, if the College do not retrace its steps ; and this is the more probable, as it seems to us a modification of some such institution must be erected upon the ruins of the Apothecaries' Act. The splendid income and large expenditure of the College will indeed wofully diminish, if the number of its admitted become confined to those whose intention it is to practise *pure surgery*—a retribution for the frequent anxious avowal by the Council of non-connexion with the great mass of their members, who are contaminated in their eyes by practising pharmacy or midwifery.

Providing a restrictive penal clause, such as we have suggested, be introduced into this Bill, we must consider the repeal of the *Apothecaries' Act,* contemplated by it, as a boon conferred upon the Profession ; and we are at a loss to account for the extravagant encomia now so freely lavished upon that measure. The conjunction that has been affected between the Worshipful Company and its quondam most bitter vituperators is intelligible enough, upon the principle, that in a death-struggle one must not be too particular in rejecting allies, come they from whence they may : but we are alluding to resolutions passed at meetings of respectable practitioners, who have never indulged in vulgar invective or unjust aspersions. That the Society has done all in its power to work well this most defective Act we willingly allow, and that it has contributed to the advancement of the condition of the General Practitioners cannot be denied; but to ascribe to it all, or even the greater portion, of their progress is to reason *post hoc propter hoc* with a vengeance. The advancement of the general practitioner has only been simultaneous with that of other branches of the profession, favored by no such tutelary power, and with the progress of science and art, not only in England, but throughout the civilized world, from the period it has been in the enjoyment of peace. "The Act of 1815, which first recognized apothecaries as legitimate practitioners was," says the Quarterly Review, "not the cause, but the consequence of the change which had taken place in their condition." And we have no hesitation in stating that this Act has of late been obstructive to farther progress; and that had a better licensing tribunal existed, both the scientific and practical attainments of the mass of practitioners would have, ere this, reached a still higher point.

Whether we consider the nature of the education enjoined, or of the examination the candidate is submitted to, we see much that calls for amendment. A servitude of five years to a practitioner, and the early age at which candidates are admitted for the license, render the preliminary education, which most who are desirous of the elevation of our profession maintain the importance of, short and unsatisfactory. Such education would be best ensured by the abolition, or great curtailment, of apprenticeship, and the admission of the licentiate a year or two later than at present; for examiners cannot otherwise expect to discover in the youth what he has had neither time or opportunity of acquiring. The Latin examinations at the Hall, for example, are a complete farce, youths who have never seen a Latin grammar, getting up, by the aid of a translation or grinder, their Celsus or Gregory, after their arrival in London, when their whole attention should be absorbed by their medical studies. Then, again, the attending the enormous number of Lectures which the Hall directs implies a physical impossibility of devoting sufficient time to the dissecting-room and hospital wards—*i. e.* the most valuable sources of knowledge. Often have we sighed with regret in observing the industrious student passing hour after hour upon the benches of a theatre, noting and afterwards revising his lectures, while living specimens of every variety of disease, and opportunities for the study of the natural and altered structure of the frame, were passed heedlessly or hurriedly by. Every hospital physician and surgeon will testify how small is the proportion of students who devote a sufficient time to hospital duties. Then, worst of all, and as a necessary consequence of this mode of pursuing study, there is the unpractical and uncertain character of the examinations. It is a growing conviction among students, that these are no fair test of their qualifications; and the necessity which well-informed and most industrious students find themselves in to be *ground* into the routine answers they will be expected to give, is a significant sign of the necessity of change. When a man who has studied under no other teacher than the *grinder* passes muster, and the laborious student, who believed himself to be above the necessity of resorting to him, is rejected, it naturally becomes a subject of inquiry upon what basis does this system rest, and why is it continued? The Examiners are not chosen from among the licentiates at large on account of manifesting fitness for a difficult avocation, but by seniority from among members of the Company. They are frequently engaged in active and fatiguing duties, are in some instances somewhat advanced in years, and in few or any are their names before the profession as cultivators of medical science or contributors of practical facts. A curious tribunal, indeed, to decide upon the claims of youths just fresh from the lecture-rooms and hospital wards, where the latest discoveries in science and improvements in practice are in course of teaching! Is it surprising that the student complains he hears opinions broached in the examination-room which he had long thought exploded, or that the *grinder* should flourish just in proportion as what is called the strictness of the examination is increased? After all the trouble taken to obtain it, the Certificate of the Hall is never looked upon by the student as a mark of distinction, or to be coveted for aught else than its allowing him to practise; while the Diploma of the College of Surgeons, for which a less laborious attendance on lectures, &c. is exacted, and a far less severe examination is endured, is regarded by its possessor as an honour. It is so, because he feels his examination has been practical, and conducted by men of great eminence in that branch of the profession, and has been passed, that is to say if he has been moderately industrious, without the degrading aid of the grinder.

We repeat, we do not mention these circumstances as condemnatory of the Society of Apothecaries, whose Act crippled their will and power to be serviceable. Indeed, it can only be the conviction of their having done as well as it permitted them, that has allowed the long continuance of the anomaly of the great body of general practitioners deriving their right to practise from a trading corporation, which possessed no claim for taking the lead in so momentous a matter. The General Practitioners who associated for the protection of their interests in 1812, had a far better idea of the requirements of their class, than those have who now propose a mere modification of the Apothecaries' Act. They proposed the licensing tribunal to be formed of Physicians, Surgeons, Apothecaries, and General Practitioners; and a board so constituted, and equitably adjusted, seems to us to afford the best chance for ensuring the efficiency and respectability of the examination. The confining the examination of the practitioner to his own grade, as recommended by the "Manifesto," we think would be most injudicious. Engaged in the active duties of the profession, men of eminence of this class have little time to devote to the cultivation of its scientific departments, and are even sometimes ill-informed as to its actual progress. Admirably fitted for the more practical portion of the business, they should call to their aid, from the schools and hospitals, those who, from being engaged in the business of teaching, are best able to test the progress the student has effected, and the use he has made of the opportunities he has been invested with. For furnishing their quota of this examining body, as also for supplying a head to this portion of the Profession, some description of Incorporation of the General Practitioners, quite independently however of the Society of the Apothecaries, governed by its elective Council, and represented in the General Council of Health and Education, seems essential.

The provision in the proposed Bill, for a general superintendence of the Education and Examination of candidates by the Council, is excellent, and has been long needed. The expediency, and justice too, of allowing the man who has passed his examination before, and paid for the license or diploma of any of the licensing bodies, to practise in any part of the United Kingdom he sees fit, but *without any additional fee or charge,* must be obvious.

To allude to the pamphlets mentioned at the head of this article. The two first, as we have stated, deserve a careful perusal, especially Mr. Green's, in reference to the imparting a more scientific bearing to the Profession, which he justly thinks so desirable. The "Statement" and "Address" of the Society are in every body's hand, but will fail to convince, we believe, the great body of the Profession of the expediency of replacing this body at its head. The "Manifesto" is a sturdy denunciation of the Bill, and a demand for a separate incorporation, which it seems to us it would be the wiser course to seek to engraft on to the amended Bill. The Speech of Dr. Hastings and Letter of Mr. Carter are temperate exposures of the alterations which are necessary to render the Bill satisfactory. Dr. Davies' "Exposition" contains a succinct account and criticism of the Laws which have hitherto governed the Profession, and in the Appendix an Analysis, or rather an Eulogium, of Sir James Graham's Bill, which it would seem, from his account, is perfection itself—a sentiment in which he will find few to participate.

The title of Dr. Thomson's *brochure* is not the most happy. We *repudiate* our wives—but not till after legal marriage: we *repudiate* our debts—but not till after they have been fairly contracted. But if Sir James Graham once rivets the chains of his Bill on the Profession, they will find it no easy matter to *repudiate* afterwards. No: *oppose* the Bill as much as possible, for we do not consider you have the power to repudiate or reject it, just because you do not like it. The Government is so strong, that they could carry a Bill authorizing every third birth to be destroyed, like a kitten, in the nearest pool or stream. Let not the Profession think themselves omnipotent, or they may awake, some fine morning, and find that the Home Secretary has been up before them. We are old enough to remember the *effervescence* produced from one end of the isle to the other, by the agitation among the "Surgeon-Apothecaries" prior to 1815—the memorable millenium of the General Practitioners! The Medical Reform Bill of that year was hailed with almost as much exultation as the Parliamentary Reform was in 1832. Now both reforms are trodden under foot, execrated—and REPUDIATED!

We suspect the author of this pamphlet is "clean daft." It appears that he addressed a memorial to Sir J. Graham, last year, in which the following passage occurred, and which is considered so important as to be reproduced in the present brochure.—

"Thus Education has to proclaim to mankind, that of all punishments inflicted upon civilized humanity, the most tremendous are inflicted by what is called the medical profession: that medical men, as permitted, authorized, and protected by the laws, usages, and ignorance of Governments, corrupt, torture, murder more helpless, unsuspecting victims, than a permanent cholera could ever reach; for every ten destroyed by war, medical men sacrifice twenty. Education looks forward with horror to the additional terrific incubus about to be immediately inflicted by Sir J. Graham."

The author is in a great rage with Sir James Graham for having apparently neglected his memorial. We are ourselves a little surprised that the Home Secretary should have forgotten so delectable a specimen of dogmatism! We apprehend it could hardly be paralleled in the "Annals of Medicine," which title he gives to his present memoir. We are inclined to the opinion that, were there not a single drug or a single doctor in existence, the rate of mortality would be little, if at all, increased. For if we endeavour to form some estimate of the number of people who are destroyed by quacks—by quacking themselves—and by the mistakes of the rash and the inexperienced, we shall be very likely to conclude that these contingencies pretty nearly balance the account against the number saved by regular advice. This, however, is a very different calculation from that which Dr. Thomson has formed.*

* We omitted to observe in its proper place in this article, that the present Bill is farther defective, as containing no provision for the Education, examination, and representation of the Chemists aud Druggists, and for the examination of persons practising Midwifery. All these matters should be included in one comprehensive scheme of "Medical Reform."

